# Actual Amount Adsorbed as Estimated from the Surface Excess Isotherm

**DOI:** 10.1021/acs.langmuir.3c02597

**Published:** 2024-01-12

**Authors:** Seishi Shimizu, Nobuyuki Matubayasi

**Affiliations:** † York Structural Biology Laboratory, Department of Chemistry, University of York, Heslington, York YO10 5DD, United Kingdom; ‡ Division of Chemical Engineering, Graduate School of Engineering Science, Osaka University, Toyonaka, Osaka 560-8531, Japan

## Abstract

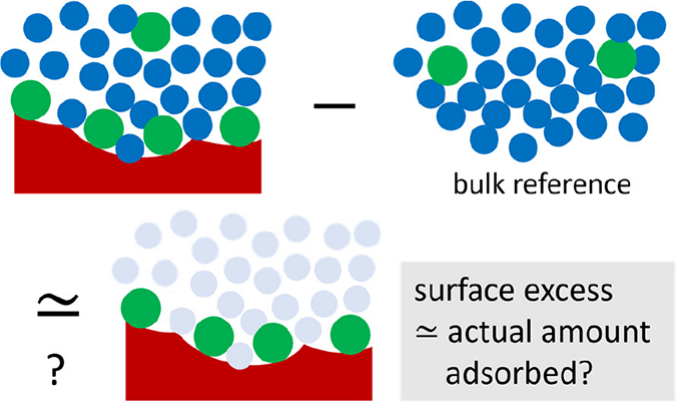

The amount of adsorption at equilibrium is commonly used for reporting solid/solution isotherms, despite the admonishment by the International Union of Pure and Applied Chemistry (IUPAC) against equating the surface excess (i.e., the measurable quantity for sorption, signifying the competitive sorption of adsorbate and solvent) with the actual amount adsorbed. The consensus, more generally stated, is that the surface excess cannot be divided into individual isotherms for sorbate and solvent unless simplifying model assumptions are introduced. Here we show, contrary to the IUPAC report, that there exists a simple method for assigning the total isotherm to the sorbate’s actual amount adsorbed and to the individual solute isotherm. This requires a combination of isotherm and volumetric measurements. For dilute sorbates, we establish criteria to show if the total isotherm is dominated by the amount of sorption at the interface, in agreement with the common assumption in the practical literature. In the absence of the volume data, we propose an approximate yet more versatile method based on the specific surface area to carry out order-of-magnitude analysis to examine whether the actual amount adsorbed dominates surface excess. Application of our methods to the adsorption of sodium decyl sulfate on polystyrene latex, malachite green on activated carbons, and thiophenes on a metal–organic framework all demonstrated the dominance of the actual amount adsorbed, significantly simplifying isotherm analysis in terms of the underlying interactions (i.e., surface-sorbate and net self-interactions at the interface), eliminating the need for excess surface quantities. Analysis of fully miscible solvent-sorbate isotherms (e.g., the mixtures of organic solvents adsorbed on mesoporous silica and carbonaceous adsorbents) indicates the contributions from both sorbate and solvent isotherms.

## Introduction

The vapor/solid isotherms are measured by the amount of adsorbates at the interface.^1−3^ However, adsorption from liquid solutions is more complex. Adsorption from solution by solids is quantified by an isotherm, which, according to the IUPAC recommendation, is “the specific reduced surface excess [...] as a function of the equilibrium liquid mole fraction”.^4^ For simplicity, let us restrict our discussion to the adsorption from binary solutions on solids. Even in this simplest case, competitive adsorption of adsorbate and solvent must be considered at the solution/solid interface.^4−6^ This is captured by the specific reduced surface excesses, denoted by IUPAC by Γ_2_^(*n*)^, that “are often referred to as composite isotherms to distinguish them from so-called ‘individual’ isotherms which purport to give the adsorption of each component separately”.^4^ According to the IUPAC report, the individual isotherms can “only be calculated on the basis of some model of the interfacial region, and have no place in the primary presentation of experimental data”.^4^

The standard practice, however, is at odds with the IUPAC recommendation.^4^ Recent reviews regarding the applications of adsorption isotherms (e.g., to pollutants,^7^ contaminants,^8^ heavy metals,^9^ wastewater treatment,^10^ and metal–organic frameworks,^11^ with a few exceptions like nanocellulose^12^) adopt *q*_*e*_ (the actual amount adsorbed at equilibrium^7,9,13,14^) instead of the reduced surface excess Γ_2_^(*n*)^ as has been recommended by the IUPAC.^4^ They implicitly assume Γ_2_^(*n*)^ ≃ *q*_*e*_, equating the surface excess with the “actual amount adsorbed”^13,14^ (i.e., the individual isotherm for sorbate), against the admonishment of the IUPAC. This can be justified for a “very dilute” solute which “is very selectively adsorbed”,^13,14^ in the framework of the “surface phase model”.^4^ The validity of such an assumption, however, has not been examined quantitatively.

The first aim of this paper is to challenge the consensus, held to this day,^5^ that individual isotherms (hence, the actual amount adsorbed) cannot be evaluated without introducing a “model of the interfacial region”.^4^ We will show how individual isotherms can be calculated when a reduced surface excess is combined with volumetric measurements. Such a possibility is well within the limits of thermodynamic principles, as can be seen from a simple calculation. A three-component system forming two phases, according to the Gibbs phase rule,^15,16^ has *F* = 3 – 2 + 2 = 3 degrees of freedom. Even when we keep the temperature constant (as has been done for isotherms), two degrees of freedom still remain. Keeping the pressure constant, while changing the sorbate chemical potential, leads to the isotherm (i.e., the reduced surface excess).^17^ However, there is another possibility: changing the pressure while keeping the composition constant (i.e., volumetric measurements), which gives complementary information on sorption.

The above strategy for evaluating individual isotherms is analogous to the solvation of biomolecules in liquid solutions,^18−20^ in which the competitive solvation of solvent and cosolute is referred to as preferential solvation.^21,22^ The mathematical analogy between preferential solvation and the Gibbs isotherm had been assumed,^22−25^ yet without any rigorous foundation, for a long time.^18^ Preferential solvation was modeled by a competitive binding of solvent and cosolute molecules on uniformly distributed binding sites on biomolecular surfaces.^25,26^ Such a primitive model was incapable of capturing crowding and steric exclusion.^27,28^ This led to the controversial misinterpretation of osmolyte exclusion as hydration.^18−20^ However, our work, which has furnished the rigorous foundation (based on the Kirkwood–Buff theory of solutions^29−31^) for preferential solvation, not only resolved the confusion and controversies caused by the misattribution^32−34^ but also established a method for evaluating both solvent-biomolecule and cosolute-biomolecule interactions (that were, before then, presumed to be inseparably linked as preferential solvation^32−34^) by complementing preferential solvation with volumetric measurements.^18−20^

Thus, our approach to achieving our first aim will be founded on the rigorous mathematical analogy between preferential solvation and the Gibbs isotherm.^29−31^ This analogy operates at a deeper level; i.e., their derivations are based on the Gibbs–Duhem equations for the system and bulk references.^18,19,30,35^ Separating a surface excess, thereby determining the individual isotherms or actual amount adsorbed, will be achieved by extending our previous work on preferential solvation^18,19,35^ to adsorption from solution, by exploiting the mathematical analogy between the two.^36,37^

The second aim of this paper is to overcome the widely acknowledged restrictions in analyzing sorption from solution by solids. IUPAC’s intended “definitive summary of the basis upon which an understanding of the phenomenon of adsorption is founded”^4^ excludes from consideration (i) “the penetration of the adsorbate into the structure of the adsorbent (e.g. swelling of clay minerals) and adsorption into swollen gels”^4^ and (ii) “adsorption from solutions of strong electrolytes, ion exchange processes, and polymer adsorption”.^4^ Both (i) and (ii) pose difficulties to the Gibbs adsorption isotherm as approached from a traditional perspective.

Here we show how our statistical thermodynamic approach has removed the restrictions one by one and how this approach can be extended further. Our first step was the generalization of the Gibbs isotherm to interfaces with arbitrary geometry. This was achieved by adopting (a) the algebraic approach to sorbent number conservation between the interfacial system and the bulk reference systems^38−40^ in place of the geometrical introduction of the dividing surface^41^ and (b) the definition of the interfacial free energy without an explicit reference to the surface area (which is difficult to define for porous systems).^40^ Our second step was to incorporate sorbate dissolution and penetration into the sorbents;^17^ we have shown that the penetration or dissolution of sorbate does not affect the basic relationships of the fluctuation sorption theory and the isotherm equations derived from it.^17^ This was achieved by the rederivation of the Gibbs isotherm from a pair of the Gibbs–Duhem equations (for the interfacial system and the bulk solution), analogous to the statistical thermodynamic preferential solvation theory.^17^ The key idea was adopting the sorbent insolubility condition as the alternative for the Gibbs dividing surface.^40^ These two steps have led to the elimination of (i) and (ii) in the previous paragraph. Based on these achievements, our second aim of the present paper is to show that strong electrolytes as sorbate can naturally be handled by the fluctuation sorption theory and its isotherms.^40,42−44^

## Theory

### Adapting the Gibbs Isotherm for Volumetric Measurements

Surface tension measurements at higher pressures have been carried out for gas/liquid and liquid/liquid interfaces.^45−49^ However, high-pressure measurements are difficult to perform for solid/liquid interfaces.^50^ Instead, density and dilatometry measurements have been carried out as the alternative (and thermodynamically equivalent) route.^51−53^ Such measurements are inevitably restricted to particles, such as polystyrene latex, for which density measurements are possible for evaluating their volume.^50,53−56^ This experimental setup will be taken into consideration in the theoretical discussion below.

Here we present an approach to the Gibbs isotherm that is better suited to volumetric measurements. Let us consider a three-component system consisting of sorbent (species *e*), solvent (1), and sorbate (2). The system forms two phases: sorbent and solution phases, denoted as the superscripts *I* and *II*, respectively. (The entire system is denoted by *.) In this framework, instead of explicitly introducing the adsorption layer, sorption is described as the net excess number of sorbates from the bulk solution.^40^ In the standard approach, the Gibbs isotherm is derived from a trio of Gibbs–Duhem equations for *, *I*, and *II* (see Supporting Information: Section A. The Generalized Gibbs Isotherm).^6,19,37,41^ Instead, for reasons that will be made clear in the next subsection, here we consider a pair of Gibbs–Duhem equations for the entire system (*) and the reference solution phase (*II*) as^17^
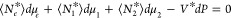
1a
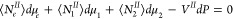
1bwhere ⟨*N*_e_^*^⟩, ⟨*N*_1_^*^⟩, and ⟨*N*_2_^*^⟩ are the numbers of sorbent, solvent, and sorbate in the system, ⟨*N*_*e*_^*II*^⟩, ⟨*N*_1_^*II*^⟩, and ⟨*N*_2_^*II*^⟩ are the numbers of the corresponding species in the reference solution phase, V* and *V*^*II*^ are the volumes of the system and reference solution phase, *P* is the pressure, and *μ*_*i*_ is the chemical potential of species *i*. Note that * and *II* have been defined in exactly the same way as for the conventional approach. Here, we introduce only one assumption: the sorbent molecules do not dissolve into the solution phase (i.e., phase *II*), which translates to^17^
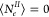
1cThis is the alternative for the Gibbs dividing surface condition in our present formalism (as will be shown below). Under this condition (eq 1c), subtracting eq 1b from eq 1a yields
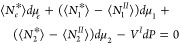
2where *V^I^* is the volume of the sorbent phase. We have shown in Supporting Information: Section B. Generalization to Strong Electrolytes that eqs 1a–1c holds for strong electrolyte sorbate and sorbent when their chemical potentials, *μ*_*e*_ and μ_2_, are taken as the sum of ionic species (*μ*_*e*_ = *μ*_*ea*_ + *μ*_*ec*_ and μ_2_ = μ_2*a*_ + μ_2*c*_, where *a* and *c* denote anion and cation, respectively). Now we introduce
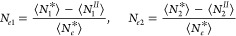
3aas the surface excesses of species 1 and 2. Their units, per unit mass of sorbent (⟨*N*_e_^*^⟩), are in line with experimental convention. We have also used
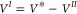
3bfor volume conservation. Using eqs 3a and 3b, eq 2 can be rewritten as
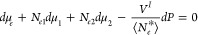
3cUnder constant pressure, taking the μ_2_-derivative of eq 3c yields
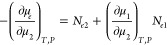
4aUsing the bulk relationship (eqs 1b and 1c), we obtain
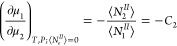
4bwhere *C*_2_ is the sorbate/solvent mole ratio in the bulk. Combining eqs 4a and 4b led to a well-known relationship:
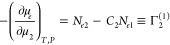
4cThus, we have obtained the relative surface excess, Γ_2_^(1)^, from the pair of Gibbs–Duhem equations (eqs 1a and 1b) under the insoluble sorbent condition (eq 1c) as an alternative for the dividing surface. As is well-known, Γ_2_^(1)^ can be obtained from the experimentally accessible reduced surface excess, Γ_2_^(*n*)^ via Γ_2_^(*n*)^ = *x*_1_Γ_2_^(1)^ (where *x*_1_ is the mole fraction of the solvent).^4^

### Determining Individual Isotherms

Now we return to our fundamental question. Is the surface excess divisible? Can the individual isotherms, *N*_*e*1_ and *N*_*e*2_, be determined independently? Evaluation of *N*_*e*1_ and *N*_*e*2_ cannot be done by the isotherm (eq 4c) alone. Determining two unknowns (i.e., *N*_*e*1_ and *N*_*e*2_) requires two inputs. This necessitates a complementary relationship independent of eq 4c. Such a relationship can be derived from eq 3c, by taking its pressure derivative, as
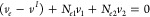
5awhere *v*_*e*_ and *v*_*i*_ are the partial molar volumes, that are the pressure derivatives of the respective chemical potentials,^15,16^ via
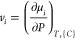
5bfor *i* = 1, 2, *e*, where {*C*} signifies keeping all the mole ratio constant. *v*^*I*^ is the volume per unit mass of the sorbent phase, defined as
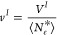
5cSolving eqs 4c and 5a as a pair of simultaneous equations will yield *N*_*e*1_ and *N*_*e*2_, the individual isotherms, as its solution, as
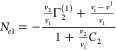
6a
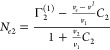
6bThe appearance of *v*_*e*_ in eq 6a and 6b rationalizes why we adopted the pair (not trio) of the Gibbs–Duhem equations (eqs 1a and 1b) as our starting point. This is because volumetric measurements can determine *v*_*e*_ of the sorbent, such as polystyrene latex. Note that *v*_*e*_ is usually measured in terms of volume/mass, which means that ⟨*N*_e_^*^⟩ has the units of mass, and *N*_*ei*_ is measured per unit mass of the sorbent.

Here we provide useful relationships, especially for strong sorption at low concentrations. It has been assumed that Γ_2_^(1)^ for strong sorption is dominated by *N*_*e*2_ even though this widely held belief has not been examined quantitatively due, perhaps, to the presumed inseparability of Γ_2_^(1)^.^4^ This can be achieved by eliminating *N*_*e*1_ from eq 4c via eq 6a, leading to
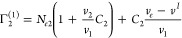
7Here, the volumetric data for *v*_1_, *v*_2_, and *v*_e_ can be compared to the surface excess (Γ_2_^(1)^), to examine how dominant the contribution from the individual sorbate isotherm, *N*_*e*2_, is.

Thus, we have achieved our first aim, by establishing how individual isotherms can be calculated through a combination of the experimental isotherm and volumetric measurements.

### Sorption of Strong Electrolytes

The difficulties regarding the application of the Gibbs isotherm to strong electrolytes have long been recognized, including (i) electric double-layer formation and (ii) ion exchange at the interface. These factors have necessitated careful considerations, including the introduction of the Gibbs dividing surface. Here we demonstrate how our alternative formulation (eqs 1a–1c) can circumvent such difficulties (Supporting Information: Section B. Generalization to Strong Electrolytes).

First, the Gibbs isotherm analogue (eq 4c) involves the insoluble sorbent condition (eq 1c) as the alternative for the Gibbs dividing surface and its geometry-free algebraic generalization. As long as the quantities of sorbent and solution are known, the Gibbs–Duhem equations (eqs 1a and 1b) can uniquely be written down, without any need for introducing the dividing surface. Such a theoretical setup is in line with the experimental reality, which can be appreciated most effectively by rewriting eq 4c into the following form:
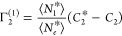
8where *C*_2_^*^ = ⟨*N*_2_^*^⟩/⟨*N*_1_^*^⟩. Equation 8 means that Γ_2_^(1)^ can be evaluated from (a) the sorbent/solvent ratio and (b) the change of the sorbate/solvent ratio upon the introduction of sorbent. All it measures is the net deviation from the bulk value of the solvent-sorbate difference around the sorbent. Neither in experiment (eq 8) nor theory (eq 4c) does the precise location of the dividing surface become a necessary piece of information. Such a formalism needs no alteration for the electrolyte sorbates except for the introduction of the sorbent chemical potential as the sum of constituent ionic species (see Supporting Information: Section B. Generalization to Strong Electrolytes).

Second, the dissociation of the sorbent at the interface does not violate eq 1c, because the ions dissociated from the sorbent form an electric double layer and are located in the vicinity of the interface. Consequently, there is no change necessary to the Gibbs–Duhem equation for the bulk solution (eq 1b) and to the insoluble sorbent condition (eq 1c), except for introducing the effective sorbent chemical potential as the sum of constituent ionic species, as has been shown in Supporting Information: Section B. Generalization to Strong Electrolytes.

Thus, we have shown that the Gibbs isotherm analogue (eq 4c) is applicable even to strong electrolytes, thereby achieving our second aim.

## Results and Discussion

### Strategy

Here we apply our theory to the combination of isotherm and volumetric measurements available in the literature. We emphasize that our analysis will be limited by the extreme rarity of the experimental volumetric data pertaining to solid/solution interfaces with varying sorbate concentrations.^50,53−56^ From the rare collection of published data, we found the volumetric data on polystyrene latex particles in aqueous surfactant solutions by Vignola et al.^56^ The limitation of the available data makes it impossible to evaluate *N*_*e*1_ and *N*_*e*2_ directly via eqs 6a and 6b. Consequently, our focus will be to examine whether Γ_2_^(1)^ ≃ *N*_*e*2_ is accurate, as has been assumed without consideration by many modern practitioners.

To facilitate the comparison, let us employ the published isotherm model constants, such as the Langmuir model, the most commonly applied for adsorption from solution, which has the following functional form:
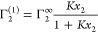
9awhere Γ_2_^∞^ is the saturating value for Γ_2_^(1)^, *K* is commonly referred to as the Langmuir constant, and *x*_2_ is the mole fraction of sorbates in the solution phase.^57^ Note that Γ_2_^(*n*)^ ≃ Γ_2_^(1)^ for dilute sorbates. Based on our recent work which (i) showed that the Langmuir model is the special and restricted case of the statistical thermodynamic ABC isotherm and (ii) provided the physical interpretations of the Langmuir model parameters,^57^*K* has acquired a new statistical thermodynamic interpretation: the difference in sorbate and solvent self-association between the bulk and the interface.^57^ Noting that *C*_2_ ≃ *x*_2_, eqs 7 and 9a in combination can be rewritten as
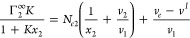
9bConsequently, the dominance of *N*_*e*2_ in Γ_2_^(1)^ can be established by

Condition I: 

Condition II: 

Condition III: *Kx*_2_ ≪ 1

Note that Conditions II and III not only lead to a mathematical simplification of eq 9b but also are consistent with the common experimental practice focusing on the low *x*_2_ region. Thus, establishing the dominance of the individual isotherm for sorbate (*N*_*e*2_) can be achieved straightforwardly by comparing the published Langmuir parameters with the volumetric data. (Note that this approach can be extended straightforwardly to other isotherm equations if necessary.)

Satisfying Conditions I–III establishes
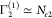
10aas has been aimed (i.e., to show the determinability of the individual isotherm, *N*_*e*2_, through the combination of isotherm and volumetric data). Satisfying these conditions also leads to
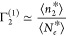
10bwhere ⟨*n*_2_^*^⟩ is the amount of sorption; hence, Γ_2_^(1)^ can simply be interpreted as the amount of sorption per unit sorbent mass (see Supporting Information: Section C. Surface Excess and the Amount of Sorption for derivation). This is exactly what has been assumed, without proof or justification, by the practitioners. Thus, we have established a trio of quantitative criteria (I–III), by which, when satisfied, Γ_2_^(1)^ can be identified as what the practitioners call *q*_*e*_, the amount of adsorption at equilibrium.

### Generalization to the ABC Isotherm

Recently, we have shown that the Langmuir model is a special and restricted case of the statistical thermodynamic ABC isotherm with a wider applicability.^57^ The ABC isotherm has the following functional form:^57^
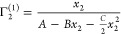
11awhich can be reduced to the Langmuir model (eq 9a) via
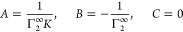
11bHere,
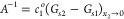
11csignifies the sorbate-surface preferential interaction over solvent-surface, defined in terms of the difference between the surface-sorbate (*G*_*s*2_) and surface-solvent (*G*_*s*1_) Kirkwood–Buff integrals, with *c*_1_^*o*^ being the molar concentration of the bulk solvent.^57^*B*, defined as
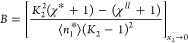
11dsignifies the difference in the Kirkwood–Buff χ parameter between the interface (χ*) and the bulk (*χ*^*II*^), signifying the net self-interaction (sorbate-sorbate *G*_22_ and solvent-solvent *G*_11_ minus sorbate-solvent *G*_12_) in the interface (*) and bulk (*II*), via^57^
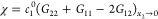
11ealongside *K*_2_ = *C*_2_^*^/*C*_2_^*II*^ being the interface/bulk partition coefficient of the sorbate. The AB isotherm has advantages over the Langmuir model.^57^ First, unlike the Langmuir model, the AB isotherm is valid for uniform site-specific (i.e., the Langmuir model) and nonspecific nonuniform sorption mechanisms alike.^57^ Second, unlike the Langmuir model, the same parameters, *A* and *B*, are shared with the statistical thermodynamic cubic isotherm which can model the IUPAC U- and S-shaped isotherms^4^ observed for completely miscible sorbate-solvent systems.^57^

In the framework of the ABC isotherm, the conditions for the dominance of *N*_*e*2_ in Γ_2_^(1)^ can be generalized via eqs 7 and 11a as

Condition I: 

Condition II: 

Condition III: 

Note that Conditions II and III not only lead to a mathematical simplification of eq 9b but also are consistent with the common experimental practice focusing on the low *x*_2_ region when the ABC isotherm is applied to partially miscible sorbate-solvent systems. Even for fully miscible sorbate-solvent systems, the dominance of *N*_*e*2_ should be established for small Γ_2_^(1)^ (i.e., when *x*_2_ is small) rather than for the trivial case of large Γ_2_^(1)^. Just like in the previous subsection, when these three conditions are met, eqs 10a and 10b are satisfied.

### Volumetric Analysis

Here we demonstrate that a combination of isotherm and volumetric data allows the examination of Conditions I–III in the previous paragraph, establishing that the surface excess is dominated simply by the amount of sorption (eq 10b).

To do so, the only available system, for which the volumetric analysis has been carried out in the form usable for Conditions I and II, is the adsorption of SDS (sodium decyl sulfate) on polystyrene latex particles. Here we carry out the order-of-magnitude analysis on volumetric data based on Vignola et al.^56^ using particles with a diameter of 33.2 nm. From their density measurement, the latex volume *v*_*e*_ was evaluated as *v*_*e*_^*o*^ = 0.9736 cm^3^ g^–1^ (in pure water at 25 °C for negatively charged particles)^56^ and Δ*v*_*e*_ (the volume of transfer from water to aqueous surfactant solutions) via *v*_*e*_ = *v*_*e*_^*o*^ + Δ*v*_*e*_. The maximum value for Δ*v*_*e*_ is around 0.06 cm^3^ g^–1^ at around the CMC;^56^ hence, we chose the value, *v*_*e*_ ≃ 1.04 cm^3^ g^–1^ as its upper bound. Now we estimate *v*^*I*^, the volume of phase *I* per mass, from the literature value of the polystyrene latex density, *v*^*I*^ = 1/(1.055 g cm^–3^) = 0.948 cm^3^ g^–1^. Consequently, *v*_*e*_ – *v*^*I*^ is estimated to be on the order of 10^–2^ cm^3^ g^–1^ at the very maximum. Using the partial molar volume of water, the upper bound of (*v*_*e*_ – *v*^*I*^)/*v*_1_ is estimated to be around 10^–2^/18 ≃ 5.5 × 10^–4^ mol g^–1^.

To examine the validity of Condition I, we will use the Langmuir model parameters for the adsorption of SDS on polystyrene latex, with a narrow size distribution around 94.9 nm in diameter, by Nodehi et al.^58^ (Table 1). Even though the latex sizes used for isotherm and volumetric analyses are different, we compare them per surface area. To do so, let us convert (*v*_*e*_ – *v*^*I*^)/*v*_1_ into the same units as Γ_2_^∞^*K*. This can be achieved by using the volume-to-surface ratio of the polystyrene latex sphere *r*_*l*_/3 (where *r*_*l*_ is the radius) and the latex density *d*_*l*_ via *r*_*l*_*d*_*l*_/3, which will be used to convert the units of (*v*_*e*_ – *v*^*I*^)/*v*_1_. Table 1 carries out unit conversion so that the adsorption and volumetric data could be compared in the same units. The comparison shows Γ_2_^∞^*K* ≫ (*v*_*e*_ – *v*^*I*^)/*v*_1_, thereby satisfying Condition I.

**Table 1:** Establishing the Predominance of the Sorbate Individual Isotherm in the Relative Surface Excess via Condition I Using the Langmuir Parameters and Volumetric Data

Adsorption^a^		Volumetric	
*K*	6.69 × 10^3^	(*v*_*e*_ – *v*^*I*^)/*v*_1_	5.5 × 10^–4^ mol g^–1^
Γ_2_^∞^	7.257 × 10^–10^ mol cm^–2^	*r*_*l*_*d*_*l*_/3	5.8 × 10^–7^ g cm^–2^
*K*Γ_2_^∞^	4.85 × 10^–6^ mol cm^–2^	(*v*_*e*_ – *v*^*I*^)/*v*_1_^b^	3.2 × 10^–10^ mol cm^–2^

a The Langmuir parameters are taken from Nodehi et al.,^58^ and *K* has been transformed into mole-fraction-based.

b Estimation based on Vignola et al. via *r*_*d*_ = 16.6 nm^52^ and the unit conversion by multiplying *r*_*l*_*d*_*l*_/3.

Our next task is to examine Condition II. Due to the diluteness of the surfactant, it is natural to assume that *v*_1_ hardly changes from its pure water value. The upper bound value for *v*_2_ is around 230 cm^3^ mol^–1^ at 30.1 °C by Shinoda and Soda^59^ and about 250 cm^3^ mol^–1^ at 25 °C by Vass et al.^60^ Consequently, we adopt *v*_2_/*v*_1_ ≃ 250/18 ≃ 14 as its upper bound. This is negligibly small compared to  even at the maximum surfactant concentration, *C*_2_ ≃ 1.82 × 10^–4^. This demonstrates that Condition II is satisfied. For sufficiently small *x*_2_ (where the dominance of *N_e_*_2_ should be examined), Condition III is satisfied.

Thus, we have completed the order of magnitude analysis based on the combination of the isotherm and volumetric data for polystyrene latex particles in a water/SDS mixture. For this system, we have shown that the surface excess Γ_2_^(1)^ is dominated by the individual isotherm of SDS on the polystyrene latex, *N*_*e*2_. The individual isotherm for water contributes negligibly to the surface excess.

### Estimation via Specific Surface Area

The necessity for the rare volumetric data for establishing the dominance of *N*_*e*2_, the individual isotherm of sorbates, severely restricts the applicability of our theory. Here, we propose how the volumetric information for examining Condition I in the previous subsection can be estimated by the specific surface area, which is a common, routinely measured quantity for adsorbents. To do so, let us note that *v*_*e*_ – *v*^*I*^ signifies the volume of the interfacial layer, which can be estimated roughly by a convenient value for the surface area of the particles, combined with a reasonable value for the interlayer thickness. (The statistical thermodynamic method from our previous papers^43,44^ gives a more reliable method for the surface area, though literature values are often quoted as BET surface areas.) Let the thickness of the interfacial layer be *δ*_*l*_ and the BET surface area be *σ*_*BET*_, through which the second term of eq 9b can be estimated as
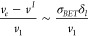
12We emphasize that eq 12 is solely for the order of magnitude estimation.

Our approach to estimation (eq 12) has been applied to the adsorption of malachite green on activated carbons by Qu et al.,^61^ who have also provided the BET surface areas of the activated carbons. Table 2, after straightforward unit conversions, shows that eq 12 is negligibly small compared to the Γ_2_^∞^*K* of eq 9b for a realistic order of magnitude for *δ*_*l*_ (which may be ∼1 nm). (To keep the calculation straightforward, we have kept the units in the original paper,^61^ which were L mg^–1^ for *K*_*L*_ for simple cancellation of mg when evaluating Γ_2_^∞^*K*.) The calculation in Table 2 shows that Γ_2_^(1)^ for malachite green adsorption is indeed dominated by the individual isotherm, *N*_*e*2_, of the sorbate when the sorbate is dilute (*x*_2_ ≪ 1, Condition II).

**Table 2:** Dominance of the Sorbate Individual Isotherm for Malachite Green Adsorption on Activated Carbons Using Specific Surface Area via Eq 12

Activated carbons	BET surface area (m^2^ g^–1^)	saturating capacity (mg g^–1^)	Langmuir constant (L mg^–1^)	σ_BET_δ_l_v_1_^–1^ (mol g^–1^)	Γ_2_^∞^K (mol g^–1^)
Coconut	1101^a^	83.06^a^	0.35^a^	6.09 × 10^–2^	1.61 × 10^3^
Coal	923^a^	74.91^a^	0.27^a^	5.11 × 10^–2^	1.12 × 10^3^
Apricot	819^a^	69.59^a^	0.23^a^	4.53 × 10^–2^	8.88 × 10^2^
Peach	793^a^	69.93^a^	0.2^a^	4.39 × 10^–2^	7.76 × 10^2^

a Taken from Qu et al.^61^

Generalizing Condition I to the statistical thermodynamic ABC isotherm (eq 11a) expands its applicability furthermore. Now not only partially miscible sorbate-solvent systems (Table 3) but also fully miscible sorbate-solvent systems (Table 4) can be examined. Table 3 compares the sorption of thiophene, benzothiophone, and dibenzothiophene from water on Cu-BTC (1,3,5-benzenetricarboxylate), a metal–organic framework.^62^ For thiophene and dibenzothiophene, *A*^–1^ is three-orders of magnitude larger than σ_*BET*_δ_*l*_*v*_1_^–1^, showing that Γ_2_^(1)^ ≃ *N*_*e*2_ at low *x*_2_, where Condition II is satisfied. For benzothiophene, *A*^–1^ is about 40 times larger than σ_*BET*_δ_*l*_*v*_1_^–1^, suggesting that Γ_2_^(1)^ is still dominated by the sorbate individual isotherm *N*_*e*2_ yet less clearly so than in the cases of the two other sorbates. For the adsorption from fully miscible sorbate-solvent mixtures on mesoporous silica (SBA-16) and carbonaceous adsorbents (XEN563 and XEN572) in Table 4,^63,64^*A*^–1^ is on the same order as σ_*BET*_δ_*l*_*v*_1_^–1^, suggesting that Γ_2_^(1)^ = *N*_*e*2_ – *C*_2_*N*_*e*1_ should be considered explicitly as the competition between sorbate and solvent surface excesses, following its original definition via eq 4c.

**Table 3:** Examining Condition I for the Adsorption of Thiophenes from Water on Cu-BTC^a^

Sorbate	*σ*_*BET*_^b^ (m^2^ g^–1^)	*A*^c^ (g mmol^–1^)	σ_*BET*_δ_*l*_*v*_1_^–1^ (mol g^–1^)	*A*^–1^ (mol g^–1^)
Thiophene	1614	2.44 × 10^–5^	8.92 × 10^–2^	40.9
Benzothiophene	1614	2.93 × 10^–4^	8.92 × 10^–2^	3.4
Dibenzothiophene	1614	2.64 × 10^–5^	8.92 × 10^–2^	37.9

a Data from Liu et al.^62^ measured between *x*_2_ = 0 and 1.8 × 10^–4^ at 293.15 K.

b The BET surface area of Cu-BTB measured by Liu et al. using a N_2_ probe at 77 K.^62^

c The parameter *A* of the ABC isotherm taken from ref (57).

**Table 4:** Examining Condition I for Sorption Data for Fully-Miscible Sorbate and Solvent

Sorbent	Solvent	Sorbate	*σ*_*BET*_ (m^2^ g^–1^)	*A*^c^ (g mmol^–1^)	σ_*BET*_δ_*l*_*v*_1_^–1^ (mol g^–1^)	*A*^–1^ (mol g^–1^)
SBA-16^a^	*n*-Octane	Ethanol	806	5.59 × 10^–4^	4.47 × 10^–2^	1.79 × 10°
SBA-16^a^	Octanol	Ethanol	806	1.89 × 10^–2^	4.47 × 10^–2^	5.29 × 10^–2^
SBA-16^a^	*n*-Octane	Octanol	806	8.96 × 10^–4^	4.47 × 10^–2^	1.12 × 10°
XEN563^b^	Ethanol	*n*-Octane	496	6.26 × 10^–2^	2.75 × 10^–2^	1.60 × 10^–2^
XEN572^b^	Ethanol	*n*-Octane	995	2.93 × 10^–2^	5.51 × 10^–2^	3.42 × 10^–2^

a Original measurements by Rockmann and Kalies.^63^

b Original measurements by Kalies et al.^64^

c Fitted to the cubic isotherm in ref (57), with the units in g/mmol.

## Conclusions

This paper aimed to resolve the discrepancy between the common practice and IUPAC recommendation when interpreting adsorption from solution. While the IUPAC emphasizes that an isotherm is essentially the surface excess that cannot be separated into the “adsorption of each component”,^4^ the practitioners interpret it as the actual amount adsorbed. For its resolution, we have shown that “individual isotherms which purport to give the adsorption of each component separately”^4^ can be determined from experimental data alone. This can be achieved by combining the surface excess (i.e., the compositional variation of the interfacial free energy) with the volumetric data (i.e., the pressure dependence of the interfacial free energy). This approach is a generalization of the preferential solvation theory, whose combination with the volumetric data (i.e., the pressure dependence of the solute chemical potential) has led to determining the individual contributions of solvent and cosolute interactions with the solute (such as biological macromolecules).^18,19,36,37^ Unlike the conventional Gibbs isotherm, our approach is applicable even to strong electrolyte sorbates, even under sorbate penetration into sorbent.

Our novel approach necessitates the revision of the classical consensus, that individual isotherms can “only be calculated on the basis of some model of the interfacial region, and have no place in the primary presentation of experimental data”.^4^ No models of the interfacial region were necessary for the determination of the individual isotherms. Instead, unleashing the remaining degree of freedom from the Gibbs phase rule was necessary, utilizing the pressure axis, rarely considered previously, to complement the isotherm.

The present paper focused on the simplest case of strong adsorption in three-component systems as proof of principle. A generalization of this approach to multiple-component systems is possible, in a manner analogous to the Kirkwood–Buff solution theory for multiple-component solutions. Such a generalization can be carried out efficiently with the help of the implicit function theorem.^37^ This enables the quantitative study of weaker sorption. Although it is challenging to conduct volumetric experiments, new knowledge and understanding of the competing effects will be gained from them. We hope that the simplicity of our analysis for extracting the new data encourages experimentalists to take on the technical challenges.

## Supporting Information Available

Calculations of the generalized Gibbs isotherm; the generalization to strong electrolytes; and the surface excess and the amount of sorption (PDF)
