# Supplementary material for: Actual Amount Adsorbed as Estimated from the Surface Excess Isotherm
Source: Langmuir. 2024 Jan 12;40(3):1666–73. doi: 10.1021/acs.langmuir.3c02597 (PMC10809752; doi:10.1021/acs.langmuir.3c02597)
Supplement: Supplementary file 1 — la3c02597_si_001.pdf [file la3c02597_si_001.pdf]

Supporting information

Actual Amount Adsorbed as Estimated from the Surface Excess Isotherm

Seishi Shimizu[1,*] and Nobuyuki Matubayasi[2]
[1]York Structural Biology Laboratory, Department of Chemistry, University of York, Heslington, York YO10 5DD, United Kingdom.
[2]Division of Chemical Engineering, Graduate School of Engineering Science, Osaka University, Toyonaka, Osaka 560-8531, Japan.

Corresponding Author: Seishi Shimizu: York Structural Biology Laboratory, Department of Chemistry, University of York, Heslington, York YO10 5DD, United Kingdom.
Email: seishi.shimizu@york.ac.uk

Table of Contents

A. The Generalized Gibbs isotherm. p.S1
B. Generalization to Strong Electrolytes. p.S2
C. Surface Excess and the Amount of Sorption. p.S3.
References. p.S3.

A. The Generalized Gibbs Isotherm

Let us consider a three-component system consisting of sorbent (species e), solvent (1), and sorbate (2). Following Gibbs,[1] an interface is defined as the difference between the system that contains an interface (denoted by $*$) and the two reference systems on the solid (denoted by I) and the solution sides (denoted by II).[2] To guarantee its applicability to interfaces with complex shapes and geometry, we take a statistical thermodynamic approach based directly on thermodynamic quantities and their Legendre transforms.[3,4] The thermodynamic functions for the grand canonical ensembles (Ω) for the system ($*$) and the reference systems I and II, are

$$\Omega^* = -PV^* + F, \quad \Omega^I = -PV^I, \quad \Omega^{II} = -PV^{II} \tag{A1a}$$

where F is the interfacial free energy, V is the volume, and the pressures (P) of the system and the reference states are set as identical.[2] We have not introduced the surface explicitly to cater to porous systems for which surface areas are hard to quantify. We have already shown in our previous papers that the combination of Legendre transform ($Y = \Omega + \mu_e N_e$ to introduce partially open ensembles closed only to e), phase equilibrium (equal μ_e in the three phases), and volume conservation ($V^* = V^I + V^{II}$) lead to[2]

$$F = Y^* - Y^I - Y^{II} - \mu_e(N_e^* - N_e^I - N_e^{II}) \tag{A1b}$$

As a coordinate-free generalization of the Gibbs dividing surface, we introduce $N_e^* = N_e^I + N_e^{II}$, which is beneficial for handling rugged and porous interfaces for which coordinate systems are difficult to define.[3] This simplifies eq A1b to

$$F = Y^* - Y^I - Y^{II} \tag{A1c}$$

The differential form of eq A1c is

$$dF = (\langle N_1^* \rangle - \langle N_1^I \rangle - \langle N_1^{II} \rangle) d\mu_1 + (\langle N_2^* \rangle - \langle N_2^I \rangle - \langle N_2^{II} \rangle) d\mu_2 \tag{A1d}$$

Note that volume and number conservation conditions have been incorporated. By taking the μ_2-derivative of eq A1d, we obtain

$$-\left(\frac{\partial F}{\partial \mu_2}\right)_{P,T} = \langle N_2^* \rangle - \langle N_2^I \rangle - \langle N_2^{II} \rangle + \left(\frac{\partial \mu_1}{\partial \mu_2}\right)_{P,T} (\langle N_1^* \rangle - \langle N_1^I \rangle - \langle N_1^{II} \rangle) \qquad \text{(A2a)}$$

which contains the surface excess of sorbate $(\langle N_2^* \rangle - \langle N_2^I \rangle - \langle N_2^{II} \rangle)$ and solvent $(\langle N_1^* \rangle - \langle N_1^I \rangle - \langle N_1^{II} \rangle)$. To evaluate $\left(\frac{\partial \mu_1}{\partial \mu_2}\right)_{P,T}$, we use the Gibbs-Duhem equation for phase II,

$$\left(\frac{\partial \mu_1}{\partial \mu_2}\right)_{T,P;\langle N_e^{II} \rangle = 0} = -\frac{\langle N_2^{II} \rangle}{\langle N_1^{II} \rangle} = -C_2^{II} \qquad \text{(A2b)}$$

where C_2^{II} is the sorbate/solvent mole ratio in the bulk. Combining eqs A2a and A2b, we obtain

$$-\left(\frac{\partial F}{\partial \mu_2}\right)_{P,T} = \langle N_2^* \rangle - \langle N_2^I \rangle - \langle N_2^{II} \rangle - C_2^{II} (\langle N_1^* \rangle - \langle N_1^I \rangle - \langle N_1^{II} \rangle) \qquad \text{(A2c)}$$

This is the generalized Gibbs isotherm applicable to any interfacial geometry, which is analogous in shape to eq 4c derived in the main text.

B. Generalization to Strong Electrolytes

Here we generalize the theory of sorption, based on the pair of the Gibbs-Duhem equations in the main text (eqs 1a and 1b), to incorporate strong electrolytes.

Electrolyte Sorbates. Here we consider strong electrolyte sorbate. This means that species 2 dissociate into cation (denoted as *2c*) and anion (*2a*).

$$\langle N_e^* \rangle d\mu_e + \langle N_1^* \rangle d\mu_1 + \langle N_2^* \rangle d\mu_{2a} + \langle N_2^* \rangle d\mu_{2c} - V^* dP = 0 \qquad \text{(B1a)}$$

$$\langle N_e^{II} \rangle d\mu_e + \langle N_1^{II} \rangle d\mu_1 + \langle N_2^{II} \rangle d\mu_{2a} + \langle N_2^{II} \rangle d\mu_{2c} - V^{II} dP = 0 \qquad \text{(B1b)}$$

Note that one sorbate dissociates into one cation and one anion, hence the numbers of cation and anion are the same. Consequently, $\langle N_2^* \rangle$ and $\langle N_2^{II} \rangle$ have been used as the numbers of ions in $*$ and II. Here we introduce the overall chemical potential of sorbate, μ_2, via

$$\mu_2 = \mu_{2a} + \mu_{2c} \qquad \text{(B2)}$$

Combining eqs B1a and B1b with B2, we obtain eqs 1a and 1b in the main text that are valid for strong electrolyte sorbates when the overall chemical potential has been introduced (eq B2). Consequently, under the dissociation equilibrium (eq B2), all the discussions from eq 2 onwards remain unchanged.

Electrolyte Sorbents. Here we generalize our theory in the main text to strong electrolyte sorbent. Let α be the fraction of sorbent (species e) that are located at the surface. They can dissociate into cation (ec) and anion (ea), whose chemical potentials are denoted as μ_{ec} and μ_{ea}, respectively, through which the electrolyte version for eq 1a becomes

$$(1-\alpha)\langle N_e^* \rangle d\mu_e + \alpha \langle N_e^* \rangle (d\mu_{ec} + d\mu_{ea}) + \langle N_1^* \rangle d\mu_1 + \langle N_2^* \rangle d\mu_2 - V^* dP = 0 \qquad \text{(B3a)}$$

where μ_e is the non-dissociated species located at the sorbent interior. Considering the dissociation equilibrium,

$$\mu_e = \mu_{ec} + \mu_{ea} \qquad \text{(B3b)}$$

eq B3a reduces to the form identical to eq 1a. In addition, since the ions dissociated from the sorbent form an electrical double layer, they are absent in the bulk solution phase (II), which leads to

$$\langle N_1^{II} \rangle d\mu_1 + \langle N_2^{II} \rangle d\mu_2 - V^{II} dP = 0 \tag{B3c}$$

which is equivalent to eqs 1b and 1c in combination. Thus, we have proven that, under the dissociation equilibrium (eq B3b), all the discussions from eq 2 onwards remain unchanged.

C. Surface Excess and the Amount of Sorption

First, we show that Condition I in the main text, or its generalization,

$$\Gamma_2^{(1)} \gg C_2 \frac{v_e - v^I}{v_1} \tag{C1}$$

in the context of eq 7, is equivalent to

$$\Gamma_2^{(1)} \gg \frac{\langle n_2^{II} \rangle}{\langle N_e^* \rangle} \tag{C2}$$

where $\langle n_2^{II} \rangle$ is the number of bulk sorbates within the interfacial layer. To show eq C2 from eq C1, let us start by identifying that $1/v_1 = \langle N_1^{II} \rangle / V^{II}$ for dilute solutions. Consequently,

$$C_2 \frac{v_e - v^I}{v_1} = \frac{\langle N_2^{II} \rangle}{V^{II}} (v_e - v^I) \tag{C3}$$

where the right-hand side signifies the number of bulk sorbates found within the interfacial volume per unit sorbent mass, $v_e - v^I$, which can be denoted as

$$C_2 \frac{v_e - v^I}{v_1} = \frac{\langle n_2^{II} \rangle}{\langle N_e^* \rangle} \tag{C4}$$

which, in combination with eq C1, proves eq C2.

Second, we show that $\Gamma_2^{(1)}$ is the amount of sorption under eq C2. To do so, let us employ the postulate

$$\langle N_2^* \rangle - \langle N_2^{II} \rangle \simeq \langle n_2^* \rangle - \langle n_2^{II} \rangle \tag{C5}$$

signifying that the predominant contribution to the surface excess (i.e., the left-hand side of eq C5) comes from the interfacial layer (i.e., the right-hand side of eq C5). With this postulate and Condition II, $\Gamma_2^{(1)}$ can be rewritten as

$$\Gamma_2^{(1)} \simeq \frac{\langle n_2^* \rangle - \langle n_2^{II} \rangle}{\langle N_e^* \rangle} \tag{C6}$$

Combining eqs C6 and C2 leads to

$$\Gamma_2^{(1)} \simeq \frac{\langle n_2^* \rangle}{\langle N_e^* \rangle} \tag{C7}$$

which justifies the unproved assumption in the practical literature.

References

(1) Gibbs, J. W. *The Collected Works of J. Willard Gibbs. Vol. 1*; Longmans, Green and Co.: New York, 1928.
(2) Defay, R.; Prigogine, I. Tension Superficielle et Adsorption; Desoer: Liege, 1966; pp 71–79.
(3) Shimizu, S.; Matubayasi, N. Fluctuation Adsorption Theory: Quantifying Adsorbate-

Adsorbate Interaction and Interfacial Phase Transition from an Isotherm. *Phys. Chem. Chem. Phys.* **2020**, *22*, 28304–28316. https://doi.org/10.1039/D0CP05122E.
(4) Shimizu, S.; Matubayasi, N. Sorption: A Statistical Thermodynamic Fluctuation Theory. *Langmuir* **2021**, *37*, 7380–7391. https://doi.org/10.1021/acs.langmuir.1c00742.
